# Visit Patterns for Severe Mental Illness with Implementation of Integrated Care: A Pilot Retrospective Cohort Study

**DOI:** 10.3934/publichealth.2015.4.821

**Published:** 2015-12-15

**Authors:** Meghan Fondow, Nancy Pandhi, Jason Ricco, Elizabeth Zeidler Schreiter, Lauren Fahey, Neftali Serrano, Marguerite Burns, Elizabeth A. Jacobs

**Affiliations:** 1. Access Community Health Centers; 2. University of Wisconsin School of Medicine and Public Health; 3. Health Innovation Program; 4. Primary care Academics Transforming Healthcare Collaborative; 5. University of Minnesota

**Keywords:** primary care behavioral health, integrated care, severe mental illness, primary care, visit patterns

## Abstract

There is increasing interest in models that integrate behavioral health services into primary care. For patients with severe mental illness (SMI), a population with disproportionate morbidity and mortality, little is known about the impact of such models on primary care clinic utilization, and provider panels. We performed a retrospective cohort pilot study examining visit patterns for 1,105 patients with SMI overall, by provider, before, and after the implementation of a primary care behavioral health model which had a ramp up period from May 2006-August 2007. We used 2003–2012 electronic health record data from two clinics of a Federally Qualified Health Center and conducted interrupted time series and chi-square analyses. During the intervention period there was a significant increase in the proportion of visits per month to the clinic for patient with SMI relative to overall visits (0.27; 95% CI 0.22-0.32). After the intervention period, this rate declined (-0.23; -0.19-0.28) but remained above the pre-intervention period. After integration of behavioral health into our primary care clinics, there was a sharp increase in the number of patients with SMI, suggesting patient willingness to explore receiving care under this model. Clinics looking to adopt the model should be mindful of potential changes in patient subpopulations and proactively manage this transition.

## 1. Introduction

Despite clear need, individuals with severe mental illness (SMI) often do not receive adequate medical care. Consequently, individuals with SMI, defined as bipolar disorders or psychotic spectrum illnesses, disproportionately suffer from medical comorbidities and their life expectancy can be reduced by up to 25 years compared to the general population.[1] Over half of these individuals have other physical illnesses in addition to their mental health concerns.[2],[3] While primary care has been referred to as the ‘de facto mental health system’ in the United States, [4],[5] literature examining visit patterns for this vulnerable group suggests individuals with SMI are less likely than the general population to use traditional primary medical care even if they have access to it.[6],[7]

In response to the challenges of adequately addressing mental health in primary care,[8],[9] interest has grown in models that formally seek to integrate these areas. However, there has been little exploration of integrated care models for patients with SMI and what has been reported in the literature is across the spectrum of integration. This spectrum ranges from care that is simply coordinated or co-located to full collaboration where mental health providers work alongside medical providers and share treatment planning within the same medical record.[8] Integrated care models have been shown to assist in decreasing symptoms of mania, increase health related quality of life, and increase the use of preventive health services.[8],[10]–[13] The most studied model of integrated care is collaborative care which involves nurse care managers providing protocol driven measurement based-care under psychiatrist supervision while working in close collaboration with primary care physicians.[9] A few studies suggest this model is associated with decreased hospitalizations for patients with bipolar disorders as compared to usual care.[9],[10]

A promising new integrated care model, primary care behavioral health (PCBH),[10] also offers patients the opportunity to receive medical and behavioral health services within the primary care setting however has not been studied specifically among patients with SMI. This model utilizes behavioral health consultants (BHCs) who are located in the same clinic space as primary care providers (PCPs). BHCs are available to provide brief interventions and immediate support to both patients and PCPs. They do not have their own patient panel and instead are available to see any PCP’s patient. While they have a few scheduled appointments each day, the majority of their time is open to receive warm hand-offs or same-day referrals from PCPs. The BHC will then go into the exam room to see the patient for brief assessment and discussion. Brief evidence-based interventions are offered based on the patient’s current functioning, and are collaborative with both the patient and the PCP. A follow-up plan is created using a step-wise approach to care. Follow-up visits may be with both the PCP and BHC or just one of those two depending on a patient’s needs. Overall, the model allows for highly integrated care that is flexible and responsive to the needs of the patient.

The PCBH model is increasing in popularity in the United States. However, it is unclear how patterns of care will change for patients with SMI once this integrated care model is adopted by a practice. A significant shift in care patterns may occur, impacting PCP panel composition and workload. Identifying potential changes in care patterns from this emerging model of primary care delivery is critical to understanding how best to structure PCP panels to ensure efficient, high quality care if workloads increase.[11],[12]

There is considerable variability in the definition of SMI, but for the purposes of this paper, SMI is restricted to psychotic spectrum disorders and bipolar disorders. Patients with this definition of SMI are at great risk for early morbidity and mortality [20] and therefore are in great need of primary care services. However, this population of patients with mental illness is least likely to access primary health care [13],[14] Caring for patients with SMI, as defined above, can be relatively time intensive given a high burden of concurrent acute, chronic and preventive health care needs. It is difficult to manage patients’ physical health conditions when mental illness is not addressed.[15]–[18] In addition, PCPs report less comfort caring for this population and limited knowledge about how to effectively do so.[8],[9] Integrated mental and primary health care can provide an opportunity to increase PCP knowledge of appropriate treatment and when needed, share responsibility for more specialized behavioral health care with a larger care team.

The purpose of this study is to describe changes in primary care clinic visit patterns for patients with SMI and the impact on size of PCP panels before and after the integration of behavioral health services into primary care. We examine this through electronic health record data from a large Federally Qualified Health Center (FQHC) in the Midwest with a well-established PCBH model. We hypothesized that visits would initially increase and then stabilize over time due to various factors including increased identification of SMI, population mobility, and exploration of the service by patients as word of mouth spread in the community.

## 2. Materials and Methods

### 2.1. Setting

This study was approved by the University of Wisconsin-Madison Health Sciences Institutional Review Board with a minimal risk waiver of consent. Data were from two Access clinic sites in Madison, Wisconsin. Access is a FQHC that provides medical and other services to over 25,000 individuals from underserved communities in Dane County each year. Characteristics of this patient population in 2013 are shown in Table 1.

These clinics were early adopters of integrated behavioral health services into primary care. Implementation of the PCBH began in May 2006 and was complete by August 2007. The model at Access has high fidelity to the original description,[10] and has been nationally recognized.[19]–[21] Continuing model fidelity is demonstrated by maintaining the focus on population-based care with an average 18–20% penetration into the medical population, and 85–90% adherence to 4 or fewer visits per patient per year.[10] The sole variation has been the addition of a consulting psychiatry service.[21]

Specific to Access, BHCs are psychologists or licensed clinical social workers. In addition, a consulting psychiatrist is available to provide recommendations regarding ongoing care, medication management options, and monitoring parameters for long-term medication use. Access to psychiatric consultation provides increased support for PCPs to manage patients with SMI. However, the PCP retains full responsibility for patients, including the prescribing of medications.

### 2.2. Data Extraction and Sample

For this pilot study, we performed a secondary retrospective analysis of electronic health record data. Encounter level claims data were retrieved from the electronic health record via its Clarity database.

Our sample included patients ≥18 years old who had at least two billable office visits at either clinic site between January 1, 2003 and December 31, 2012, with at least one of those visits including an encounter diagnosis for SMI. Given the transient nature of this population, we required two office visits in order to indicate an ongoing relationship with Access. SMI is defined as any of the following International Classification of Diseases, 9th revision, codes: schizophrenia spectrum disorders (295.0–295.9), bipolar disorders (296.0, 296.1, 296.4–296.9), delusional disorders (297.0–297.1, 297.9), or other psychosis (298.0–298.9). Since this was a retrospective analysis of electronic health record data, patients were considered enrolled into the study cohort when they met the inclusion criteria, and their primary care visit patterns were measured thereafter until 2012.

We describe visit patterns and characteristics at the patient, clinic, and provider panel levels for the patients with SMI included in our sample. For our provider panel analysis we used a subsample as follows: we identified all PCPs for the study patients and restricted our sample to PCPs who had at least 0.20 FTE and were present before and after model implementation. In order to calculate the impact of integrated behavioral health on individual provider panels, we assigned patients with SMI to a single PCP with whom they had the most billable Evaluation & Management office visits during the entire study period. For patients who saw two or more medical providers for the same number of visits, the billing provider for the last encounter before the end of the study period was designated as the PCP.

## 3. Measures

After cohort entry, all face-to-face visit types were included (both physical and behavioral health). Patient characteristics examined included age, sex, race/ethnicity, and insurance type. At the provider panel and clinic levels, we calculated the total number of visits among sample members per year over the study period. In order to account for clinic growth over the study period not necessarily related to integration of behavioral health services, we also determined the annual percentage of total visits made by patients in the study cohort at both the clinic and provider panel levels. To do this at the clinic level, we used the number of visits by cohort members as the numerator and total visits to the entire clinic as the denominator and examined this monthly. This process was repeated at the provider panel level using only the subset of patients who were assigned to these providers.

### 3.1. Analysis

All analyses were conducted using Stata 12.1. First, chi-square statistics were computed for two-way analyses of differences in the categorical demographic characteristics of patients who met the cohort inclusion criteria before and after the implementation of the PCBH model. Next, additional chi-square statistics were calculated for goodness-of-fit analysis of the year-to-year changes in the proportion of cohort to total clinic visits. We grouped individuals according to the year in which they met the study inclusion criteria and computed the extent to which each group contributed to the total SMI visits per year.

After these exploratory analyses, segmented linear regression analysis was performed to analyze visits at clinic level to estimate changes in clinic visit numbers from the pre-PCBH intervention period (February 2003 to May 2006) to the post-PCBH intervention period (August 2007 to December 2012). This approach estimates a slope for each of the time segments before and after the intervention. We excluded the 14 month phase-in period from June 2006 to July 2007 from our models. Regression modeling was performed using the Stata ITSA command. We then assessed for autocorrelation using the Cumby-Huizinga test and controlled for a second-order autoregressive correlation structure.

## 4. Results

### 4.1. Patient level findings

Overall, 1,105 patients met criteria for study inclusion and analysis (Table 1). Of these, 406 patients with SMI were enrolled in the study cohort prior to PCBH model implementation, and 699 patients were enrolled after the model was in place. Compared to those who enrolled before, patients enrolling after model implementation were significantly younger (*p* = 0.001) and male (*p* = 0.036) (Table 1). Due to a higher percentage of patients being categorized as unreported race and ethnicity among those who were enrolled after the model, differences in race (*p* = 0.046) and ethnicity (*p* = 0.008) were significant. There was not a significant difference in insurance type between those who were enrolled before and after PCBH model implementation.

**Table 1.** Access patients with severe mental illness characteristics*

		All Patients (n = 14,810)	Patients with SMI (n = 1,105)	Enrolled Pre-Behavioral Health Consultation (n = 406)	Enrolled Post-Behavioral Health Consultation (n = 699)	p-value
		**N (%)**	**N (%)**	**N (%)**	**N (%)**	
**Age, in years**						**0.001**
	0–19	4642 (31)	22 (2)	6 (1)	16 (2)	
	20–44	5548 (37)	628 (57)	204 (50)	424 (61)	
	45–64	3553 (24)	430 (39)	181 (45)	249 (36)	
	65 +	1067 (7.2)	25 (2)	15 (4)	10 (1)	
**Sex**						**0.036**
	Male	8621 (58)	478 (43)	159 (39)	319 (46)	
	Female	6189 (42)	627 (57)	247 (61)	380 (54)	
**Race**						**0.046**
	White	7563 (51)	653 (59)	242 (60)	411 (59)	
	Black/African American	3322 (22)	305 (28)	117 (29)	188 (27)	
	Asian	730 (5)	62 (6)	26 (6)	36 (5)	
	American Indian/Alaskan Native	1148 (8)	29 (4)	11 (3)	18 (3)	
	Unreported	2047 (14)	56 (3)	10 (2)	46 (6)	
**Ethnicity**						**0.008**
	Hispanic/Latino	5399 (36)	62 (6)	26 (6)	36 (5)	
	Non-Hispanic/Latino	9170 (62)	987 (89)	370 (91)	617 (88)	
	Unreported	241 (2)	56 (5)	10 (3)	46 (7)	
**Insurance type**						0.337
	Uninsured	3991 (27)	414 (37)	164 (40)	250 (36)	
	Medicaid	5885 (40)	377 (34)	126 (31)	251 (36)	
	Medicare	1453 (10)	202 (18)	76 (19)	126 (18)	
	Commercial	3481 (24)	112 (10)	40 (10)	72 (10)	
**Top 4 diagnoses for 2013**						
	Pain Diagnoses	3665 (25)				
	Acute Illness	1946 (13)				
	Hypertension	1602 (11)				
	Diabetes	1457 (10)				

*Bold values indicate significance (*p* < 0.05)

### 4.2. Clinic level findings—time series regression

**Figure 1.**
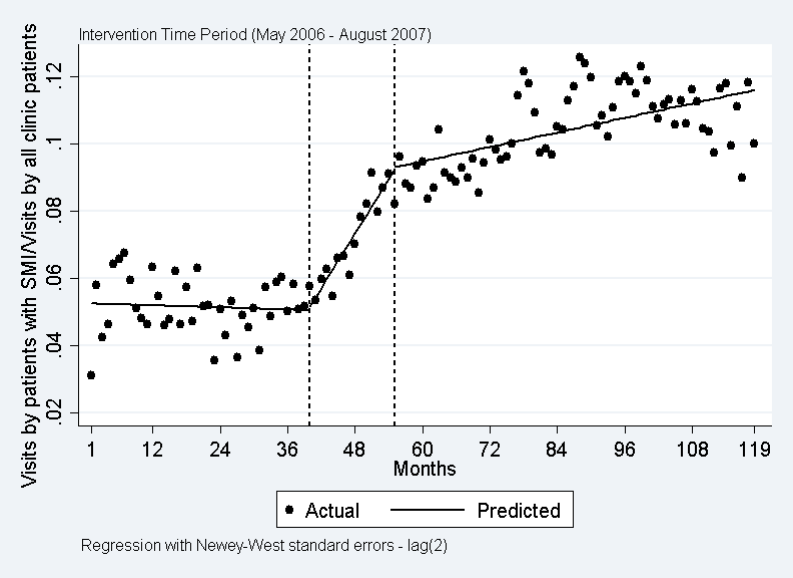
Actual and predicted values for number of visits for patients with SMI relative to total clinic visits over time.

Figure 1 shows actual and predicted values of visits for patient with SMI relative to overall visits over time. Prior to the intervention, the visit slope did not show a significant increase (-0.005; 95% CI 0.003–0.000). During the intervention period there was a significant level shift (0.27; 95% CI 0.22-0.32). After the intervention period, the visit slope declined (-0.23; -0.19–0.28) but remained above the pre-intervention period.

### 4.2. Provider level findings

**Figure 2.**
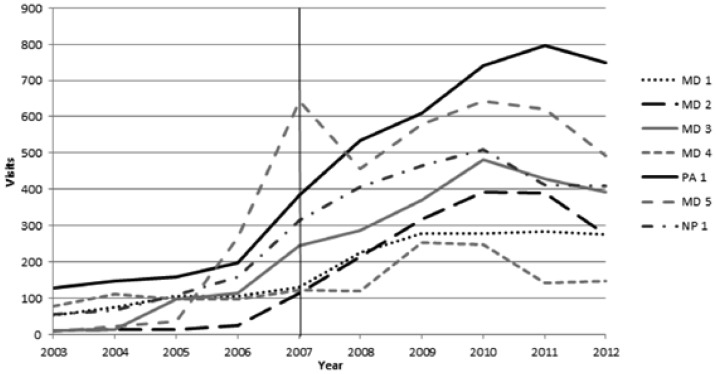
Severe mental illness patient visits per provider panel by year. MD = Medical Doctor; PA= Physician Assistant; NP = Nurse Practitioner.

Of the seven providers whose panels we followed in our sub-cohort, five were primary care physicians, one a nurse practitioner, and one a physician assistant. These providers cared for 716 patients with SMI in our cohort. Figure 2 shows visits per year for patients with SMI by these provider panels. Visits increased steeply beginning in 2007 for all provider panels, although to a variable degree, and continued to increase until stabilizing in 2010 and 2011. Of note, the nurse practitioner and the physician assistant saw both the largest increases and total numbers of visits for patients with SMI compared to the five physicians.

**Figure 3.**
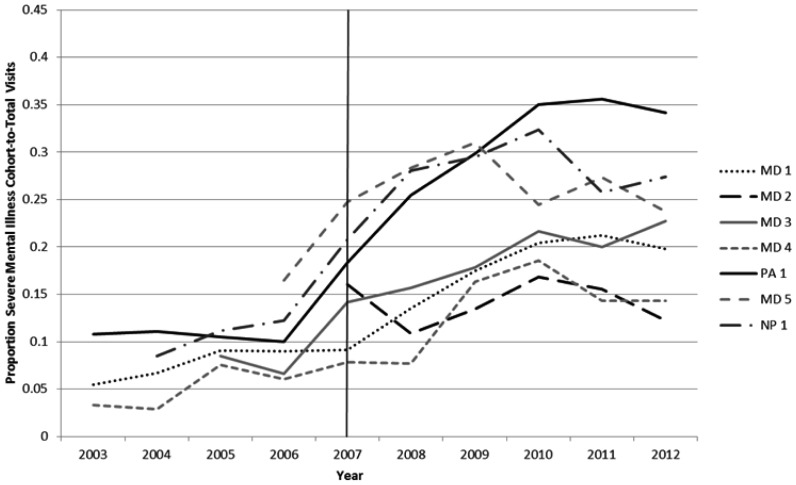
Proportion of severe mental illness cohort to total patient visits per provider panel by year. MD = Medical Doctor; PA= Physician Assistant; NP = Nurse Practitioner.

Figure 3 depicts the proportion of patients with SMI on each PCP’s panel, adjusted for the increase that occurred in total patients per panel over the study period. Again, there was a significant but variable increase in the proportion of SMI patients on all panels following model implementation. The physician assistant’s panel had the highest proportion of patients with SMI among the seven PCPs. The proportion of patients with SMI on provider panels peaked in 2010–2011 and then began to decrease.

**Figure 4.**
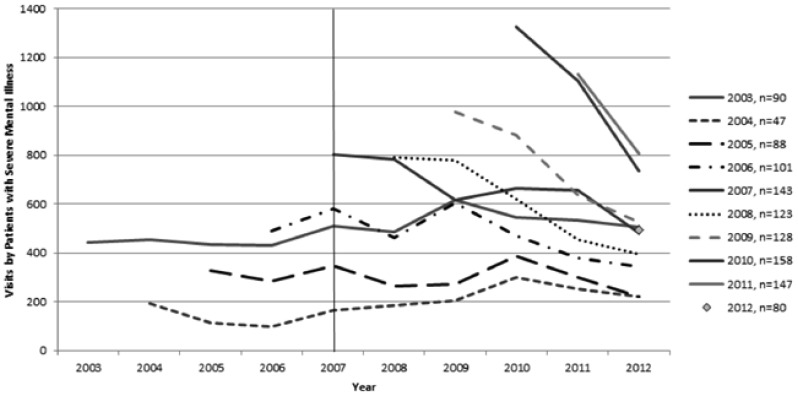
Number of visits by patients with severe mental illness per year by cohort.

The individual contributions to visits by cohorts based on year of study enrollment are illustrated in Figure 4. This figure demonstrates that the majority of the increased visits after model implementation came from patients who entered the cohort around the time of model integration or in the couple of years afterwards (those meeting study inclusion criteria in 2007–2011). Patients with SMI who entered the cohort prior to model implementation had overall stable visit patterns over the study period.

## 5. Discussion

We found that after integrating behavioral health services into primary care, both the annual number of visits and proportion of total clinic visits made by patients with SMI increased. Overall, the percentage of visits by this cohort compared to total clinic visits increased sharply during model implementation. As hypothesized, there was a pattern of increased visits and then later stabilization. This pattern was consistent across individual provider panels within the subset analyzed, but providers varied widely in the proportion of visits occurring for patients with SMI. New patients with SMI establishing care likely accounted for the increase in visits during model implementation.

To our knowledge, this is the first study to depict visit patterns for patients with SMI and the impact on provider panels before and after integration of PCBH model into primary care. Our findings suggest that an increasing number of patients with SMI will seek care in the primary care setting with the addition of integrated behavioral health. The increase in visits implies that this population is at least initially curious about the option of obtaining care within this model. Therefore, integrating behavioral health care into primary care may be a solution to the disproportionate medical comorbidity burden and current lack of primary care usage in this vulnerable population.[6],[7] We also found that the overall percentage of visits for patients with SMI increased initially for individual providers, but appeared to stabilize with time. Given interest in structuring PCP panels to allow for efficient and quality care, our results are reassuring in that they imply that the burden on providers from a SMI population after implementing an integrated model is not ever-increasing.

We found that while all providers showed increased visits by patients with SMI after model implementation, there was marked variation among providers in the proportion of panel visits occurring by this population. This variation may be due to differences in providers in terms of interest and/or comfort in caring for this group or the quality of their interactional style.[6],[7] Provider interest and comfort are important to consider when implementing integrated care models, and these areas were an explicit focus for the Behavioral Health Consultation team at Access. The Behavioral Health Consultation team regularly provides trainings for clinic staff on working with various patient populations. Provider interest and comfort are also likely to evolve as they work within the integrated care model alongside the BHCs and successfully care for this population.

The initial increases in visits for patients with SMI during the intervention period that we observed may imply that this group of patients is more willing to engage in care if an integrated model is in place. It also may reflect that the providers were more willing to provide care for this population with the assistance of the Behavioral Health Consultation team, including consulting psychiatry. The subsequent drop-off may represent an element of patient self-selection; some patients may prefer traditional specialty care over care integrated within primary care. It will be important for clinics contemplating implementing PCBH or similar integrated care models to consider the impact of increases in the representation of patients with SMI in their population. They should also set clinic policies that assist in establishing guidelines for whose behavioral health care can be appropriately managed within primary care. However, it is also important that these policies remain flexible and are revised frequently, as provider knowledge and comfort with patients with SMI are likely to increase as the model is implemented.

Our study has several limitations. First, generalizability may be limited, given that it occurred in an environment where there are shortages in specialty behavioral health care, including psychiatric care. It is possible that other clinics located in communities with greater access to psychiatric care would not see the same dramatic increases in new patients with SMI after model integration. In addition, we also were unable to identify whether the pattern of visits could be explained by patients becoming more stable and therefore needing less care, seeking care elsewhere, or simply leaving the area. Interestingly, a recent study of the collaborative care model for mental health treatment within primary care also found that mental health care contacts decreased over time.[22] Furthermore, we were unable to distinguish between visits that occurred primarily to address a physical health concern (e.g., a cold) as opposed to a behavioral health concern. Lastly, this study primarily is descriptive, and so causality cannot be directly inferred. Further research as to the influence of behavioral health integration into primary care is needed.

In conclusion, we observed increased utilization of primary and behavioral health care by patients with SMI during the implementation of an integrated care model within primary care. While model implementation may have placed an initial increased burden on providers, this increase in visits was transitory and stabilized over time. Continued research to demonstrate why these visit pattern changes occurred is critical to assist clinics in planning for implementation of integrated behavioral health services and to continue to increase access to primary and behavioral health care for this population. In particular, further research also is needed regarding long-term effectiveness of engaging this population in health care using this integrated model, and to elucidate the impact of increased engagement on medical comorbidities and mortality. In addition, research exploring the potential economic impact of increased primary and behavioral health care for this vulnerable group of patients is needed. Despite the need for further research, our current findings will be useful for the growing numbers of practices that are interested in integrating behavioral health services into primary care.
